# Tiny Habits^®^ for Gratitude-Implications for Healthcare Education Stakeholders

**DOI:** 10.3389/fpubh.2022.866992

**Published:** 2022-05-16

**Authors:** Joshua C. Hollingsworth, David T. Redden

**Affiliations:** Edward via College of Osteopathic Medicine–Auburn, Biomedical Affairs and Research, Auburn, AL, United States

**Keywords:** gratitude, habits, Tiny Habits, self-care, COVID-19, stress, burnout, healthcare professionals (HCPs)

## Abstract

The COVID-19 pandemic has led to diminished sleep and increased stress, anxiety, and burnout for many health professionals and health professions students. One simple approach that may be effective for bolstering personal well-being is consciously cultivating gratitude. Gratitude is positively associated with physical health, psychological health, hope, sleep, and health behavior engagement; and randomized studies indicate that gratitude interventions can improve psychological well-being and sleep. The primary aim of this study was to assess the impact of practicing Tiny Habits^®^ on self-reported gratitude, as measured by the 6-Item Gratitude Questionnaire (GQ-6). In January 2021, 154 adult participants with GQ-6 <35/42 were randomized to one of 3 groups: Tiny Habits for Gratitude (*n* = 50), Tiny Habits Control (*n* = 52), and Inactive Control (*n* = 52). Both Tiny Habits groups chose 3 Tiny Habits Recipes to practice daily and participated in the free, email-based 5-Day Program with automated daily check-in emails and personalized feedback from a Certified Tiny Habits Coach. The Recipes for the Tiny Habits for Gratitude group focused on cultivating gratitude, while those for the Tiny Habits Control group did not. Post-intervention, the mean change in GQ-6 scores in the Tiny Habits for Gratitude (Δ = ↑6.9 ± 5.6; *n* = 37/50, 74%; *p*< *0.001, Cohen's d* = *0.85*) and Tiny Habits Control (Δ = ↑5.6 ± 4.1; *n* = 31/52, 60%; *p* = *0.009, Cohen's d* = *0.71*) groups were greater than that of the Inactive Control group (Δ = ↑2.5 ± 4.4; *N* = 42/52, 81%). At 1 month, the mean change in GQ-6 scores in the Tiny Habits for Gratitude group (Δ = ↑7.0 ± 5.3; *N* = 28/50, 56%) was greater than that of the Inactive Control group (Δ = ↑2.9 ± 5.4; *N* = 39/52, 72%; *p* = *0.002, Cohen's d* = *0.78*). These findings suggest that practicing Tiny Habits Recipes and participating in the 5-Day Program can significantly increase gratitude in the short term and focusing specifically on gratitude during this process can sustain the increase in gratitude for up to 1 month. Implementation is quick, simple, and free. This has significant implications for healthcare education stakeholders.

## Introduction

Recent societal and environmental events have negatively impacted the health and wellness of many health professionals and health professions students. This is well illustrated by the impact of the COVID-19 pandemic on healthcare education stakeholders across the globe. For healthcare professionals, particularly those working on the frontlines, the pandemic brought about a significant increase in work demands, uncertainty regarding best treatment practices, emotional exhaustion from caring for severely ill patients and consoling families who have lost loved ones, and heightened concern regarding contracting and further spreading the disease themselves. The result for many has been diminished sleep and increased stress, anxiety, and burnout (1–14). Due to COVID-19-related restrictions, the requisite shifts from in-person to online learning, and the resulting decrease in social support and interaction, similar negative effects have been seen in health professions students (15–20). In addition to the negative impact of these stressors on health professionals, there are negative implications for patient safety as well (21). While effective institution- and policy-based approaches to mitigate the negative impact of such events are worth exploring, implementation is often slow and complex. In the meantime, there are actions that individuals can take to bolster their personal well-being and resiliency in the face of such challenges. One simple approach that may be effective for many is to practice and cultivate gratitude.

Gratitude can be defined as “a life orientation (i.e., worldview) toward noticing and appreciating the positive in the world” (22). It is experienced when one recognizes that (1) they have obtained a positive outcome and that (2) there is an altruistic external source (e.g., another individual, god, nature, happenstance) that is responsible for the positive outcome (23). Gratitude can be conceptualized as a state of being, a personality trait, an emotion, an attitude, or a coping mechanism (23). When viewed as a state of being, gratitude is more context-based; it is felt more during specific events. In contrast, gratitude as a trait refers to how often and intensely one experiences the state of gratitude (24). Levels of state and trait gratitude vary from person to person and often fluctuate within an individual over time (24, 25).

Higher levels of gratitude are associated with enhanced well-being and prosocial behavior. Cross-sectional studies have found that gratitude is positively associated with self-reported physical health (26), psychological health (26–28), hope (29, 30), sleep quality and quantity (31, 32), and engagement in health behaviors (26). A few studies have assessed gratitude and related outcomes in healthcare education stakeholders. Looking at nurses in South Korea (*N* = 646) during the COVID-19 pandemic, Lee et al. (33) found that gratitude was negatively associated with perceived stress. Prior to the pandemic, a study by Shi and Du (34) involving 1,392 medical students in China found that gratitude was positively associated with perspective taking and empathic concern, two components of emotional intelligence which have been found to be positively associated with prosocial behavior (35). This link between gratitude and prosocial behavior is corroborated elsewhere in the literature (28).

Systematic reviews and meta-analyses of randomized studies indicate that gratitude interventions provide meaningful health and wellness benefits. A meta-analysis by Davis et al. (36) indicated that gratitude interventions, such as keeping a daily or weekly gratitude journal or expressing gratitude to others, had a small positive effect on psychological well-being. Cregg and Cregg (37) found in their meta-analysis a modest effect of gratitude interventions in terms of reducing symptoms of depression and anxiety when assessed post-intervention and up to 1 month thereafter, and a systematic review by Boggiss et al. (38) suggested that gratitude interventions improve subjective sleep quality. Lastly, in a series of meta-analyses, Dickens (39) found that gratitude interventions provide many positive benefits with small to medium effect sizes regarding well-being, happiness, and depressive symptoms when assessed post-intervention and at delayed follow-up (ranging from 1 week to 6 months). It should be noted that the outcomes that were found to be significant, as well as the corresponding effect sizes, depended upon the comparison group. As a general trend, benefits and effect sizes were smaller when gratitude interventions were compared to a positive control (e.g., listing daily acts of kindness), larger when compared to a negative control (e.g., listing daily hassles), and somewhere in between when compared to a neutral control (e.g., measurement only or listing daily activities) (36, 37, 39). The benefits of gratitude interventions outlined above focus on comparison to neutral control groups.

A few mechanisms have been proposed regarding the beneficial effects of gratitude on psychological well-being and sleep. Fredrickson's (40, 41) broaden-and-build theory posits that gratitude, like other positive emotions (e.g., joy, love), broadens one's “momentary thought-action repertoire” and builds one's “enduring personal resources.” In other words, positive emotions enable individuals to pursue a wider range of novel or creative thoughts and actions in the moment (e.g., prosocial or reciprocal altruistic behaviors), resulting in an increase in one's social resources (e.g., more friendships and social bonds) and psychological resources, such as hope (30) and resilience (33). Building on Fredrickson's model, Alkozei et al. (42) suggest that, by increasing the experience of positive memories for past events, gratitude broadens one's ability to interpret seemingly negative or ambiguous situations with a more positive valence, thereby promoting psychological well-being. This is in opposition to negative emotions (e.g., fear, anxiety) which narrow one's momentary thought-action repertoire, promoting reflexive and adaptive responses (e.g., fight or flight) to threatening situations (40, 41). As for sleep, a cross-sectional study by Wood et al. (32) with over 400 participants found that gratitude predicted better subjective sleep quality and sleep duration, and less sleep latency and daytime dysfunction. Mechanistically, the authors proposed that gratitude mediates the relationships observed by promoting positive vs. negative pre-sleep cognitions.

In order to be a viable option for healthcare education stakeholders, simple, attractive, and effective gratitude-boosting interventions are needed. Several interventions, such as regularly writing a gratitude list (“counting one's blessings”), keeping a gratitude journal, or writing and delivering a letter of gratitude to an individual, have been shown to boost gratitude (39). Given that healthcare education stakeholders are often overworked with little time, physical energy, or mental energy to spare, particularly during the pandemic (9), effective interventions that are quick and easy to perform are needed. Further, interventions that individuals are intrinsically motivated to perform would lend themselves to habit formation and increased engagement (43). Although existing interventions that are supported in the literature are relatively easy and take little time to perform, the options are limited, and it is possible to create and test interventions that are simpler still. The ideal intervention is one that is effective and appealing to the individual, can be tailored to fit seamlessly into their personal routine, takes minimal time and energy to perform, and allows for easy revision, as needed, in the face of barriers or changes in routine.

The Tiny Habits^®^ Method (44) is a simple, systematic and evidence-based approach to designing new behaviors into one's existing routine. The Tiny Habits Method was created by BJ Fogg, PhD *via* derivation from the Fogg Behavior Model (45). The Method involves creating a specific form of implementation intentions (46) called Tiny Habits Recipes, as follows: “After I Anchor Moment, I will Tiny Behavior.” The Anchor Moment is some reliable behavior in one's existing routine that is used to prompt the new target behavior. The Tiny Behavior is the target behavior of interest but scaled back, if needed, such that it takes very little time (i.e., <30 s) and effort to perform. Here's an example Recipe related to gratitude: “After I brush my teeth in the morning, I will think of one thing for which I am grateful.” Immediately after the Tiny Behavior, a planned Celebration is performed. The Celebration can be anything that the individual can think, say, and/or do in the moment (e.g., think, “It feels great to be grateful!” and do a fist pump) to generate positive emotions and the feeling of success. The purpose of the Celebration is to reinforce the Tiny Behavior and the Anchor Moment–Tiny Behavior relationship, thereby increasing the automaticity—the defining feature of habits (47)—with which the Tiny Behavior is performed after the Anchor Moment. Practitioners of the Method are encouraged to focus on behaviors that they truly “want” to do, as opposed to those that they feel they “should” or “need to” do (44). The Tiny Habits Method is supported in the literature as follows. Implementation intentions have been shown to facilitate action and goal achievement (46) by enhancing the mental accessibility of cues (Anchor Moments) and strengthening the link between cues and planned responses (Tiny Behaviors) (48, 49). Further, previous studies show that small, simple behaviors are performed more consistently and are more readily formed into habits (47, 50), as are behaviors that are pleasurable and intrinsically motivated (43, 51).

## Materials and Methods

A randomized controlled study was performed to assess changes in gratitude scores (primary outcome) in a Tiny Habits for Gratitude group as compared to a Tiny Habits Control group and an Inactive Control group. Change in hope (secondary outcome) was also assessed, as hope and gratitude have been shown to be positively associated (29, 30).

### Sample

A power analysis was performed using PASS 14.0 (NCSS, LLC. Kaysville, Utah) to determine the number of participants needed to detect variation in group means accounting for 10% of the total observed variance. Using an alpha level = 0.05 and 85% power, analysis results indicated that a total sample size of 102 participants (*N* = 34 per group) was needed. To account for attrition, the target sample size was set at 150 participants (*N* = 50 per group).

Inclusion criteria required participants to be ≥18 years of age and to have a baseline score <35/42 on the Gratitude Questionnaire 6-Item (GQ-6), a validated gratitude assessment (28). The GQ-6 cut-point was set at <35 to avoid the ceiling effect (52).

### Assessments

The participants responded to the following assessments at three timepoints: before the intervention (time 1 = baseline), directly after the intervention (time 2 = post-intervention), and approximately 1 month after the intervention (time 3 = follow-up).

### Gratitude Questionnaire 6-Item

The GQ-6 is a validated, self-administered tool for assessing gratitude. It consists of 6 statements with responses classified on a 7-point Likert-type scale ranging from 1 = strongly disagree to 7 = strongly agree. Two of the 6 items are reverse scored. Possible scores range from 6 to 42, with higher scores signifying higher gratitude (28).

### Adult Hope Scale

The AHS is a validated, self-administered tool for assessing hope, defined as “a cognitive set that is based on a reciprocally derived sense of successful (a) agency (goal-directed determination) and (b) pathways (planning of ways to meet goals)” [(53), 571]. It consists of a total of 12 items with responses classified on an 8-point Likert-type scale ranging from 1 = definitely false to 8 = definitely true. Four of the items serve as “filler.” Scores are derived from the remaining 8/12 items and range from 8 to 64, with higher scores signifying higher hope [(53), 571].

### Procedures

The assessments were built and administered *via* Qualtrics (Qualtrics, Provo, UT), an online survey tool. The baseline assessment included age and sex, and all 3 assessments captured participants' first names and email addresses, such that individual responses could be linked across all 3 timepoints. Recruitment was performed *via* posts on social media (e.g., Twitter, Facebook) on a rolling basis. After clicking the link, potential participants first viewed an information letter detailing the study, including potential benefits as well as risks, which were minimal. Those meeting inclusion criteria were then randomized (1:1:1) to one of three groups using the “randomizer” feature in Qualtrics.

Participants who were randomized to one of the Tiny Habits groups then selected 3 Tiny Habits Recipes from a list of 10 to practice. The Recipes for both groups included the same Anchor Moments but differed on the Tiny Behaviors. The Tiny Behaviors presented to the Tiny Habits for Gratitude group focused on gratitude, while those presented to the Tiny Habits Control group did not. See Table 1. These two groups also participated in the Tiny Habits 5-Day Program (54). This free, email-based, semi-automated program includes (1) daily check-in emails with additional guidance for applying the Method and (2) personalized input and feedback from a certified Tiny Habits Coach, as needed. These participants were encouraged to practice their Recipes, revising them as needed, during the 5-Day Program and beyond. Total daily time commitment for completing the 3 recipes and fully participating in the 5-Day Program is estimated to be <2 min and <5 min, respectively. There was no intervention for the Inactive Control group. Post-intervention (time 2) and follow-up (time 3) assessments were emailed to all participants ~1 week and 5 weeks after enrollment (time 1). No incentives were offered to participants.

**Table 1:** Tiny Habits^®^ recipes for the Tiny Habits for gratitude and Tiny Habits control groups.

**Anchor Moments**	**Tiny Behaviors**	
	**Tiny Habits for Gratitude**	**Tiny Habits Control**
***After I…***	***I will…***	***I will…***
First put my feet on the floor in the morning	Say, “I am thankful for this day. Let's make it great!”	Do a forward bend stretch for 3 breaths.
Hit start on the coffee maker	Think of one person who helped make me who I am today.	Will tidy one item in the kitchen.
Take the first sip of my coffee/tea	Think of one thing that I am grateful for.	Open my book and read 1 sentence.
Turn on the water to shower/bathe	Think of one thing about my body that I appreciate.	Tidy one item in the bathroom.
Take the first bite of a meal	Gratefully think of one person who helped get this food on my plate.	Think of one word to describe the taste (e.g., salty, sweet) or texture (e.g., crunchy, soft) of that bite.
Shut the door when leaving my home	Think of one thing in nature that I appreciate.	Tell myself, “Today I will be calm, focused, and productive.”
Log onto my computer for work/school	Think of one thing that I appreciate about my job/school.	Write down my top priority/task for the day.
Finish eating lunch	Send a text of gratitude to someone in my life.	Ask myself, “What's the one thing that most needs my attention right now?”
Shut the door after arriving home	Think of one thing about my home That I am grateful for.	Pour myself a glass of water.
Lay my head on the pillow at night	think of one thing from my day that went well.	Take 3 deep, relaxing breaths.

### Statistical Analysis

Participant age, gratitude (GQ-6) scores, and hope (AHS) scores at baseline, as well as change in gratitude and hope scores from baseline to post-intervention and from baseline to 1-month follow-up, were described using sample means and standard deviations by group. Analysis of Variance (ANOVA) models were utilized to test for statistically significant differences among population means across groups for these variables, with Bonferroni *post-hoc* analysis performed as needed. The assumptions of normality of residuals and homogeneity of variance were examined with normal probability plots and residual plots respectively. Sex was described by frequency and percentage of female participants by group, and Chi square test of homogeneity was used to assess for statistically significant differences regarding sex across groups.

### Ethics Statement

This study was pre-approved by the Edward via College of Osteopathic Medicine (VCOM) Institutional Review Board (IRB; Board Reference #2020-038).

## Results

Between January 5th−22nd, 2021, 400 individuals expressed interest in participating in the study. A total of 154 met inclusion criteria, completed the baseline assessment, and were randomized to one of the three groups: Tiny Habits for Gratitude (*n* = 50), Tiny Habits Control (*n* = 52), or Inactive Control (*n* = 52). A total of 108/154 (71.0%) participants were female. Frequency of female sex did not differ statistically between groups (*p* = *0.32*). Overall participant mean years of age (±standard deviation) was 44.6 (±12.3). Examination of age by treatment group revealed a significant difference in mean years of age by group (*p* = *0.02*). *Post-hoc* examination among groups indicates that the Tiny Habits for Gratitude group (41.3 ± 12.5) was statistically lower in mean age compared to the Inactive Control group (47.9 ± 11.9; *p* = *0.01*). At baseline, overall mean gratitude (GQ-6) and hope (AHS) scores (±standard deviation) were 27.5 (±5.4) and 38.5 (±9.7), respectively, with no statistical difference between groups (*p* = *0.85* and *p* = *0.50*).

**Figure 1:**
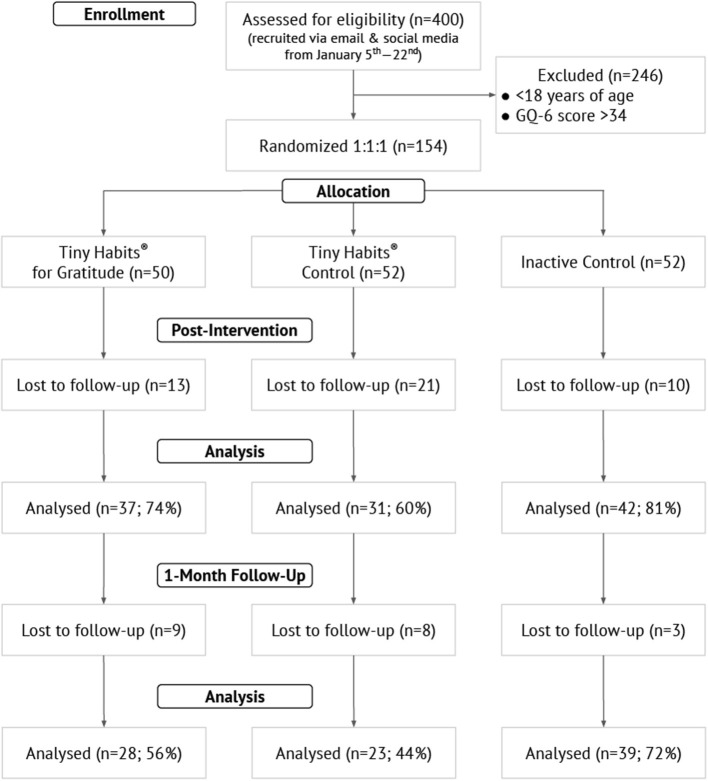
Study flow diagram.

Of the 154 participants randomized, 110 (71%) completed the post-intervention assessment and 90 (58%) completed the 1-month follow-up assessment. Comparing those who completed either the post-intervention or follow-up assessment (*n* = 116; 75%) to those who did not complete either assessment (*n* = 38), there was no statistical difference regarding mean years of age (44.9 ± 11.2 vs. 44.4 ± 14.9; *p* = 0.91) or sex (74.1 vs. 59.5% female; *p* = 0.069). Figure 1 is a flow diagram illustrating progression through the phases of the trial. Table 2 contains group-level demographic and outcomes data.

**Table 2:** Demographics and outcomes data for participants.

		**Tiny Habits for Gratitude (*n* = 50)**	**Tiny Habits Control (*n* = 52)**	**Inactive Control** **(*n* = 52)**	**Total (*n* = 154)**
**Baseline**					
Female	***n*** (% Baseline)	35 (70.0%)	33 (63.5%)	40 (76.9%)	108 (71.0%)
Years of age	**Mean** ± SD (Min–Max)	41.3 ± 12.5^a^ (18–72)	44.4 ± 11.9 (23-75)	47.9 ± 11.9^a^ (25-80)	44.6 ± 12.3 (18-80)
Gratitude (GQ-6)		27.5 ± 5.8 (10-34)	27.2 ± 5.2 (15-34)	27.9 ± 5.3 (14-34)	27.5 ± 5.4 (10-34)
Hope (AHS)		38.4 ± 11.4 (12-60)	37.4 ±9.3 (19-56)	39.7 ± 8.3 (17-55)	38.5 ± 9.7 (12–60)
**Post-intervention**					
*n* (% Baseline)		37 (74.0%)	31 (59.6%)	42 (80.8%)	110 (71.4%)
Change in gratitude (GQ-6)	**Mean** ± SD (Min–Max)	+6.9 ± 5.6^a^ (−1–+22)	+5.6 ± 4.1^b^ (−2–+16)	+2.5 ± 4.4^a, b^ (−7–+17)	+4.9 ± 5.2 (−7–+22)
Change in hope (AHS)							+9.9 ± 10.2^a^ (−5–+43)	+8.4 ± 7.5^b^ (−5–+28)	+3.6 ± 6.1^a, b^ (−10–+18)	+7.1 ± 8.4 (−10–+43)
**1-month follow-up**					
*n* (% baseline)		28 (56.0%)	23 (44.2%)	39 (72.2%)	90 (58.4%)
Change in gratitude (GQ-6)	**Mean** ± SD (Min–Max)	+7.0 ± 5.3^a^ (−1–+22)	+4.8 ± 5.3 (−9–+12)	+2.9 ± 5.4^a^ (−8–+16)	+4.5 ± 5.5 (−9–+22)
**Change in hope (AHS)**		+9.0 ± 12.4 (−12–+42)	+4.9 ± 10.2 (−20–+18)	+5.1 ± 8.1 (−9–+34)	+6.2 ± 10.2 (−20–+42)

^a^, ^b^*Indicate statistically significant differences (p < 0.05); GQ-6, 6-Item Gratitude Questionnaire; AHS, Adult Hope Scale*.

### Gratitude

**Figure 2:**
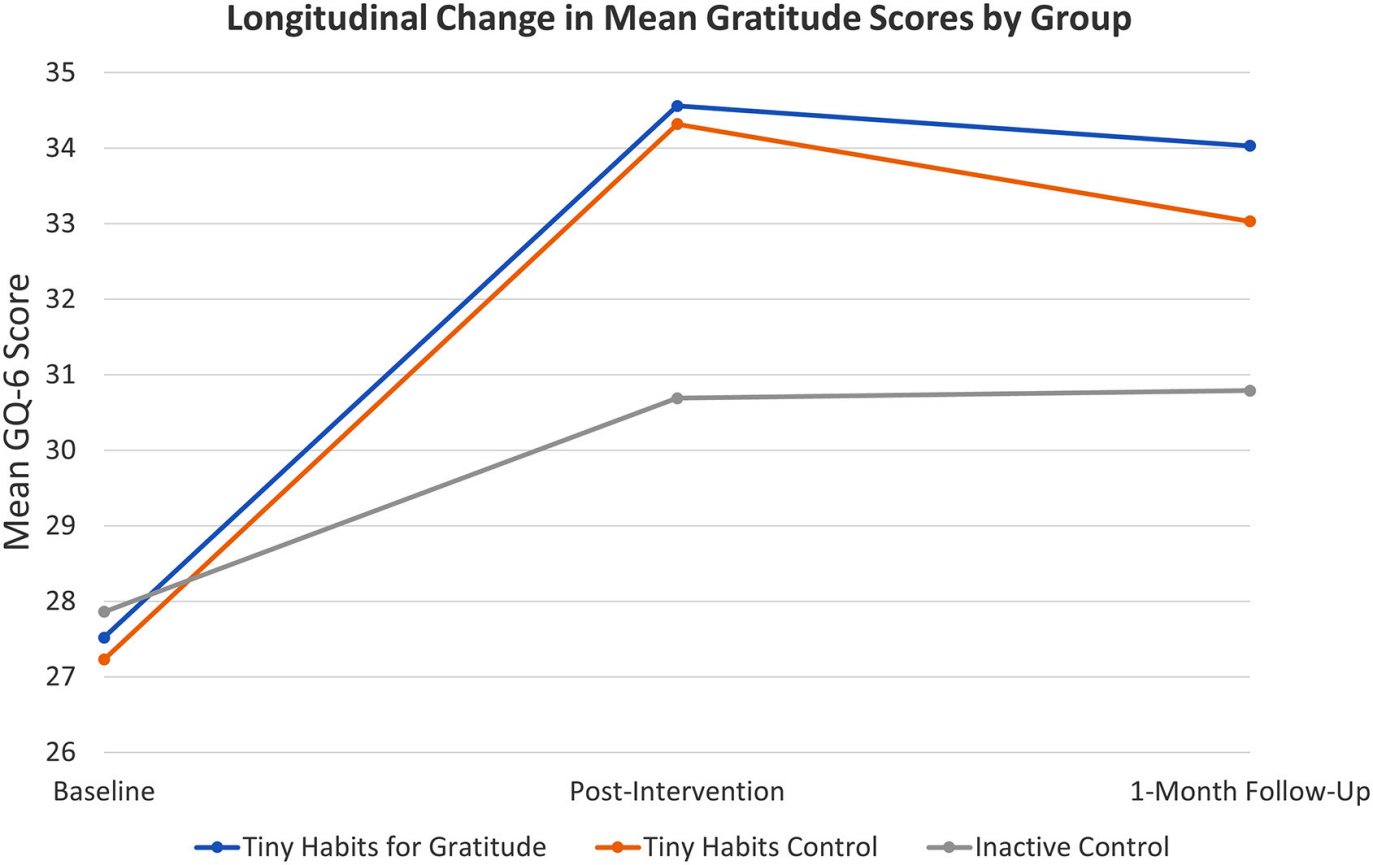
Longitudinal change in mean gratitude scores by group.

Post-intervention phase, ANOVA and Bonferroni *post-hoc* tests indicated that the mean change in GQ-6 scores in the Tiny Habits for Gratitude group (Δ = ↑6.9 ± 5.6; *N* = 37/50, 74%) and in the Tiny Habits Control group (Δ = ↑5.6 ± 4.1; *N* = 31/52, 60%) were both significantly greater than that of the Inactive Control group (Δ = ↑2.5 ± 4.4; *N* = 42/52, 81%) with *p*< *0.001* and *p* = *0.009*, respectively. Measuring the standardized difference between the sample means for Tiny Habits for Gratitude group and Inactive Control group at this time, we observe *Cohen's d* = *0.85*. Measuring the standardized difference between the sample means for Tiny Habits for Control group and Inactive Control group, we observe *Cohen's d* = *0.71*. At 1 month, the mean change in GQ-6 scores in the Tiny Habits for Gratitude Group (Δ = ↑7.0 ± 5.3; *N* = 28/50, 56%) was statistically significantly greater than that of the Inactive Control group (Δ = ↑2.9 ± 5.4; *N* = 39/52, 72%, with a *p* = *0.002* and *Cohen's d* = *0.78*. No other statistically significant differences were noted in relation to GQ-6 scores (see Figure 2).

### Hope

**Figure 3:**
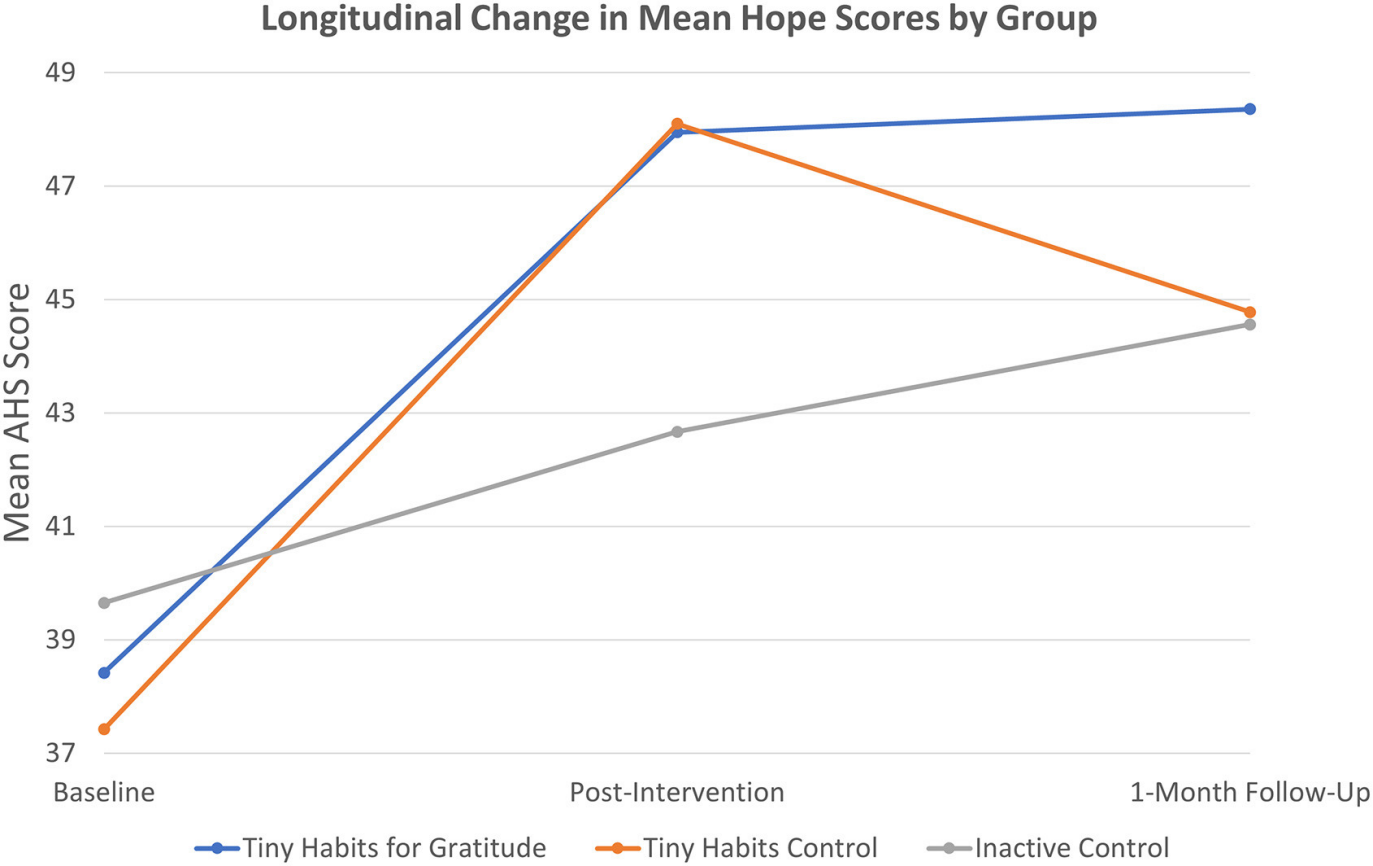
Longitudinal change in mean hope scores by group.

Post-intervention, ANOVA and Bonferroni *post-hoc* tests indicated that the mean change in AHS scores in the Tiny Habits for Gratitude group (Δ = ↑9.9 ± 10.2; *N* = 37/50, 74%) and in the Tiny Habits Control group (Δ = ↑8.4 ± 7.5; *N* = 31/52, 60%) were statistically significantly greater than that of the Inactive Control group (Δ = ↑3.6 ± 6.1; *N* = 42/52, 81%) with *p*< *0.001* and *p* = *0.015*, respectively. For these comparisons, *Cohen's d* = *0.76* comparing the Tiny Habits for Gratitude group to Inactive Control and *Cohen's d* = *0.70* comparing the Tiny Habits Control group to Inactive Control effect sizes. No other statistically significant differences were noted in relation to AHS scores (see Figure 3).

## Discussion

The findings of this study suggest that practicing Tiny Habits Recipes and participating in the Tiny Habits 5-Day Program can significantly increase hope and gratitude in the short term, and that focusing specifically on gratitude in the process can sustain the increase in gratitude for up to 1 month thereafter. Congruent with previous studies (36, 37, 39), greater differences were seen when the intervention (Tiny Habits for Gratitude) group was compared to a neutral (Inactive) vs. positive (Tiny Habits) control group. For instance, relative to the Inactive Control group, the Tiny Habits for Gratitude group exhibited statistically significant increases in gratitude scores post-intervention and at 1-month follow-up, with a large and medium-to-large effect size, respectively. And there were no statistical differences between the two Tiny Habits groups in this regard. Further, both Tiny Habits groups exhibited greater increases in hope and gratitude scores post-intervention, relative to the Inactive Control group, but only the Tiny Habits for Gratitude group exhibited sustained increases in gratitude at 1-month follow-up. This suggests that positive psychology interventions, in general, may increase hope and gratitude in the short term but, for sustained effects to be realized, such interventions may need to be focused on the domain of interest (e.g., gratitude). Overall, the results indicate that this is an effective intervention for boosting gratitude. Implementation is simple and takes very little time, and the 5-Day Program is free for all individuals who wish to take part. This has significant implications for healthcare education stakeholders, especially now.

Due to the threats and demands of the COVID pandemic, for example, many health professionals have reported diminished sleep and increased stress, anxiety, and burnout (1–14), and similar negative effects have been seen in health professions students (15–20). These effects may limit the time and physical or mental resources available to regularly engage in new behaviors (e.g., start an exercise routine, meditate, go to bed earlier) that could help promote well-being and resilience. Most individuals, however, can easily design 3 Tiny Habits Recipes for gratitude into their daily routine, with assistance from a Tiny Habits Coach in the 5-Day Program. This simple behavioral intervention can have an outsized effect in terms of boosting gratitude, which is good in and of itself and may also lead to other changes and benefits, such as improved psychological well-being and subjective sleep quality (36–39). Further, the Tiny Habits Method can also be utilized to promote consistent engagement in other gratitude interventions that have been shown to be effective. For instance, if one wanted to keep a daily gratitude journal, a Tiny Habits Recipe such as, *After I finish eating breakfast, I will open my gratitude journal and write, “Today I am grateful for __________.”* could be utilized. Increased gratitude may lead to additional health behavior change (26), and the Tiny Habits Method can be applied to any behavior domain (e.g., diet, physical activity, patient safety) of the individual's choosing, thereby providing the potential for additional benefit.

### Limitations

This study is not without limitations. The total sample size (*N* = 154) was relatively small, and a significant percentage of participants did not complete the post-intervention (28.6%) and 1-month follow-up (41.6%). Furthermore, a greater percentage of participants in the Tiny Habits groups were lost to follow-up relative to the Inactive Control group, which may bias the results. This was also an intention-to-treat analysis, in which all data available were analyzed whether or not participants completed the intervention protocol (i.e., performing their 3 Tiny Habits Recipes daily) as instructed. This is good in terms of external validity. However, completing a per-protocol analysis would strengthen internal validity. Lastly, the study was not conducted in healthcare professions stakeholders and may not generalize to this population. That said, there is no reason to believe that it would not, given the findings of previous studies regarding gratitude and healthcare professions stakeholders (33, 34).

### Future Outlooks

Practicing gratitude in general, and practicing Tiny Habits for gratitude specifically, can be a simple and effective self-care strategy to promote well-being and resilience. Future studies should consider (1) focusing on healthcare education stakeholders specifically in order to better assess the implications for this cohort and (2) incorporating per-protocol analysis to strengthen internal validity.

## Data Availability Statement

The raw data supporting the conclusions of this article will be made available by the authors, without undue reservation.

## Ethics Statement

The studies involving human participants were reviewed and approved by IRB of Edward via College of Osteopathic Medicine. Written informed consent for participation was not required for this study in accordance with the national legislation and the institutional requirements.

## Author Contributions

JH conceived of the idea, designed the study, oversaw implementation, coached participants in the Tiny Habits for Gratitude group, and drafted the manuscript. DR performed power analysis, statistical analysis, produced figures, and gave feedback on the article. Both authors approved of the article in its final form.

## Conflict of Interest

The authors declare that the research was conducted in the absence of any commercial or financial relationships that could be construed as a potential conflict of interest.

## Publisher's Note

All claims expressed in this article are solely those of the authors and do not necessarily represent those of their affiliated organizations, or those of the publisher, the editors and the reviewers. Any product that may be evaluated in this article, or claim that may be made by its manufacturer, is not guaranteed or endorsed by the publisher.
